# Restoring the perfusion of accidentally transected right gastroepiploic vessels during gastric conduit harvest for esophagectomy using microvascular anastomosis: a case report and literature review

**DOI:** 10.1186/s12893-022-01728-3

**Published:** 2022-07-28

**Authors:** Hao-Wei Kou, Pei-Ching Huang, Chon-Folk Cheong, Yin-Kai Chao, Chun-Yi Tsai

**Affiliations:** 1 Department of General Surgery, Chang Gung Memorial Hospital, Linkou Branch, College of Medicine, Chang Gung University, Taoyuan, Taiwan; 2 Department of Medical Imaging and Intervention, Chang Gung Memorial Hospital, Linkou Branch, College of Medicine, Chang Gung University, Taoyuan, Taiwan; 3 Department of Plastic and Reconstructive Surgery, Chang Gung Memorial Hospital, College of Medicine, Chang Gung University, Taoyuan, Taiwan; 4 Division of Thoracic Surgery, Chang Gung Memorial Hospital, Linkou Branch, College of Medicine, Chang Gung University, Taoyuan, Taiwan

**Keywords:** Esophageal cancer, Esophagectomy, Gastric conduit, Vascular reconstruction

## Abstract

**Background:**

Esophagectomy remains the standard treatment for esophageal cancer or esophagogastric junction cancer. The stomach, or the gastric conduit, is currently the most commonly used substitute for reconstruction instead of the jejunum or the colon. Preservation of the right gastric and the right gastroepiploic vessels is a vital step to maintain an adequate perfusion of the gastric conduit. Compromise of these vessels, especially the right gastroepiploic artery, might result in ischemia or necrosis of the conduit. Replacement of the gastric conduit with jejunal or colonic interposition is reported when a devastating accident occurs; however, the latter procedure requires a more extensive dissection and multiple anastomosis.

**Case presentation:**

A 61-year-old male with a lower third esophageal squamous cell carcinoma (cT3N1 M0) who received neoadjuvant chemoradiation with a partial response. He underwent esophagectomy with a gastric conduit reconstruction. However, the right gastroepiploic artery was accidentally transected during harvesting the gastric conduit, and the complication was identified during the pull-up phase. An end-to-end primary anastomosis was performed by the plastic surgeon under microscopy, and perfusion of the conduit was evaluated by the ICG scope, which revealed adequate vascularization of the whole conduit. We continued the reconstruction with the revascularized gastric conduit according to the perfusion test result. Although the patient developed minor postoperative leakage of the esophagogastrostomy, it was controlled with conservative drainage and antibiotic administration. Computed tomography also demonstrated fully enhanced gastric conduit. The patient resumed oral intake smoothly later without complications and was discharged at postoperative day 43.

**Conclusion:**

Although the incidence of vascular compromise during harvesting of the gastric conduit is rare, the risk of conduit ischemia is worrisome whenever it happens. Regarding to our presented case, with the prompt identification of the injury, expertized vascular reconstruction, and a practical intraoperative evaluation of the perfusion, a restored gastric conduit could be applied for reconstruction instead of converting to more complicated procedures.

## Background

Esophagectomy is a standard treatment for patients with esophageal malignancy [1]. Following esophagectomy, there are several options to reconstruct the alimentary tract, such as using the stomach, a segment of jejunum or the interpositioned colon [2]. Among them, the gastric conduit is the most commonly used substitute for reconstruction [3, 4]. The maintenance of perfusion is the keystone of the gastric conduit, which is optimized by the preservation of the right gastroepiploic artery and the right gastric artery during the harvest of the conduit. Compromising these vessels, especially the right gastroepiploic artery, might lead to the rare but devastating complication of a gastric conduit failure [4–8]. An iatrogenic injury might result from adhesions, variation of the vessels, and regional lymphadenopathy. Traditionally, jejunal or colonic interposition is the solution if the blood supply of the gastric conduit is compromised [3–5, 7, 8]. However, jejunal or colonic interposition mandates more enteric anastomosis, longer operation times, and higher morbidity rates than gastric conduits [2, 3, 9, 10]. Instead of abandoning the gastric conduit, a few studies have reported using a vascular reconstruction for a damaged right gastroepiploic vessels under this setting [11–15]. Here, we present a case of successful restoration of transected right gastroepiploic vessels using a microvascular anastomosis during esophagectomy and gastric conduit reconstruction. We have further summarized the characteristics of the vascular injury, the key surgical techniques, and clinical outcomes through this case report and present a related literature review.

## Case presentation

**Fig. 1:**
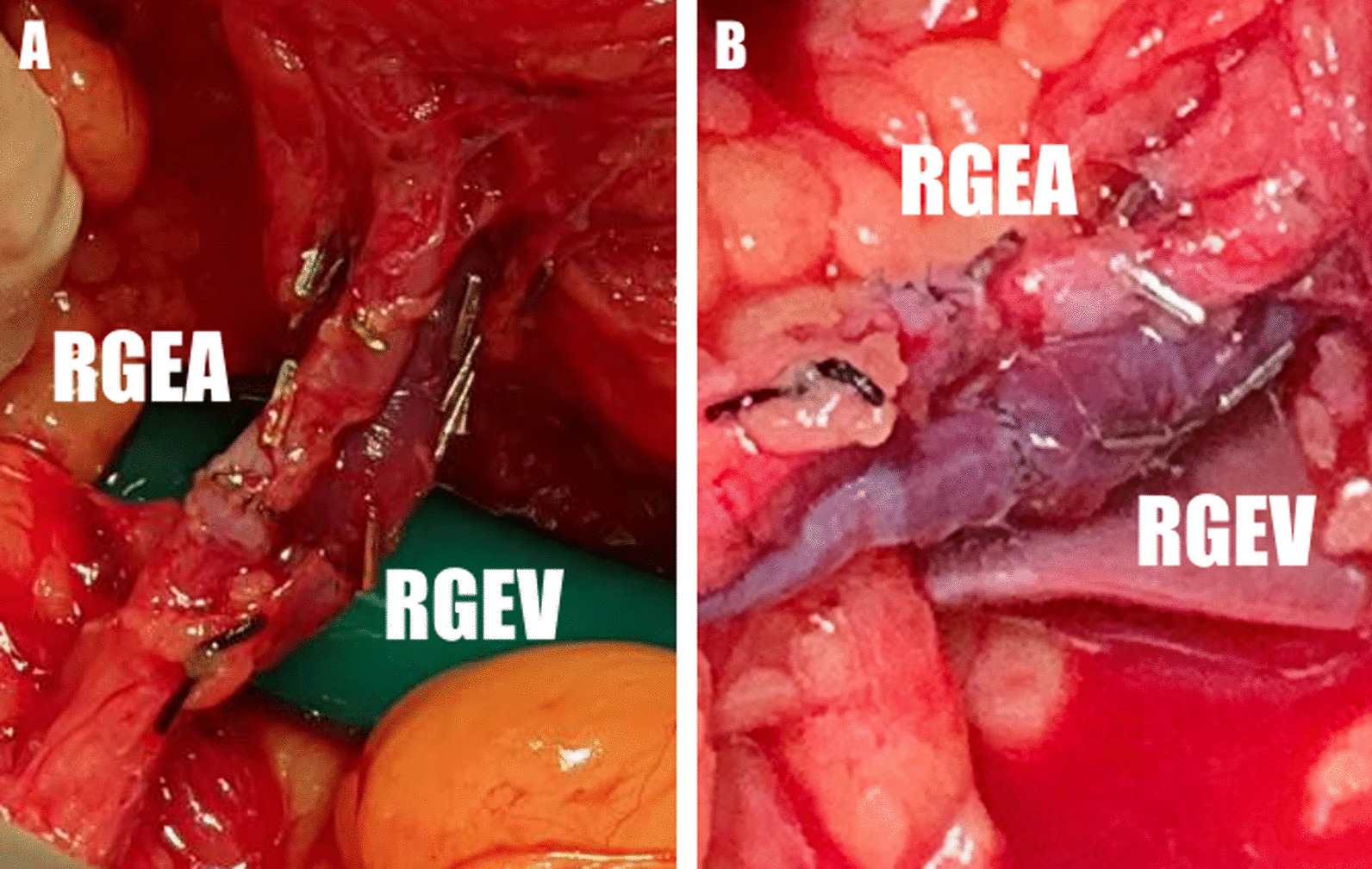
**A** After end-to-end anastomosis of the transected right gastroepiploic artery and vein. **B** In vitro view after gastric conduit pull up. *RGEA* right gastroepiploic artery, *RGEV* right gastroepiploic vein

**Fig. 2:**
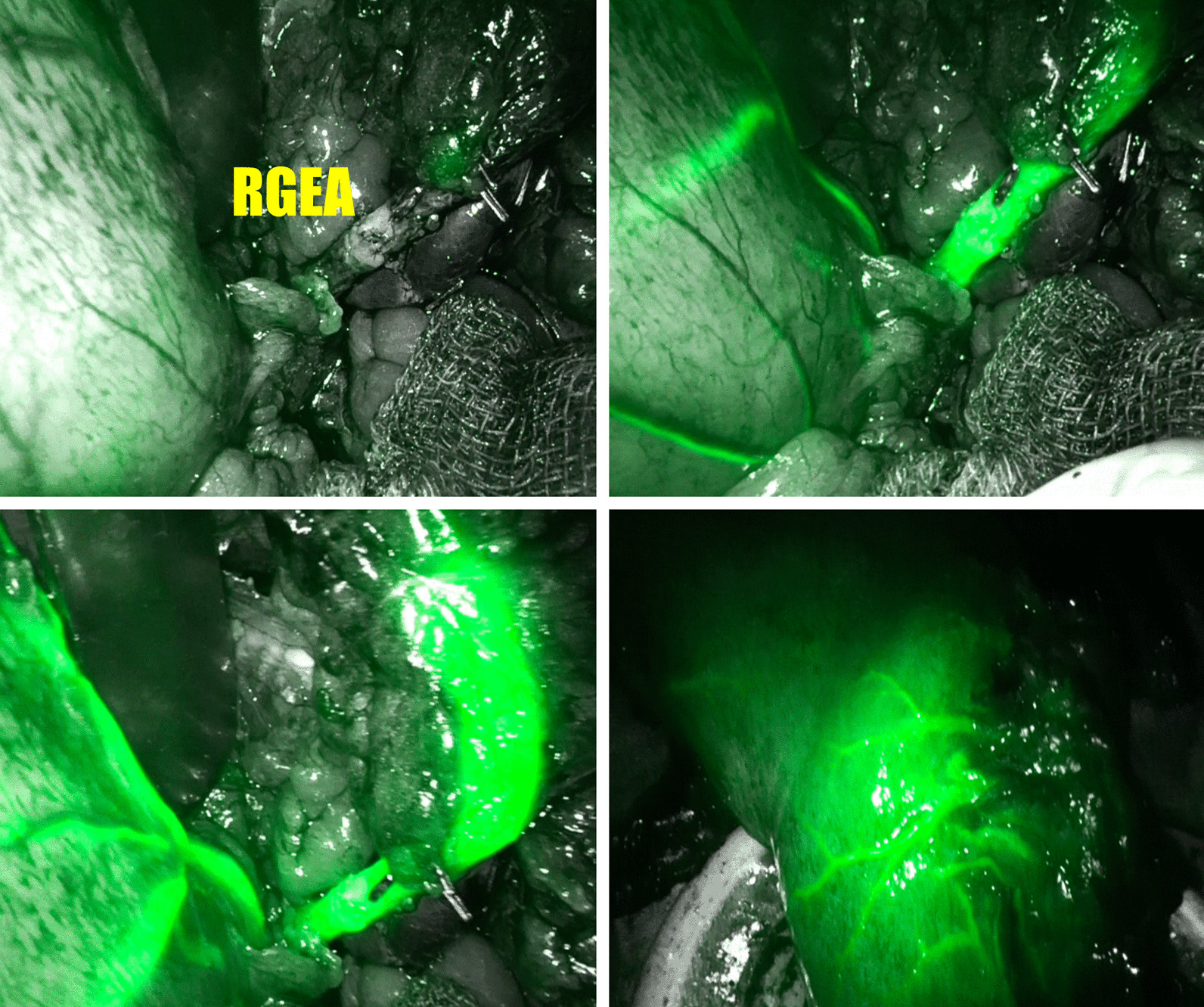
Intraoperative ICG fluorescence imaging showed patency of the reconstructed right gastroepiploic artery and adequate perfusion of the gastric conduit after vascular reconstruction. *RGEA* right gastroepiploic artery

A 61-year-old male presented with a clinical stage III (cT3N1 M0) squamous cell carcinoma at the middle third of the esophagus. After a partial clinical response following neoadjuvant chemoradiation therapy, he received a robotic assisted minimally invasive esophagectomy and reconstruction. During the laparoscopic harvest phase for the gastric conduit, the right gastroepiploic artery (RGEA) and vein (RGEV) were accidently transected and ligated by an energy device. Ischemic changes in the gastric conduit were subsequently deteced. We then converted the laparoscopic approach to a laparotomy and identified the both ends of the ligated-transected gastroepiploic vessels. End-to-end microvascular anastomosis was performed by a plastic surgeon using 8–0 nylon for both the gastroepiploic artery and vein. (Fig. 1) After revascularization, indocyanine green (ICG) fluorescence imaging was applied to evaluate the perfusion of the gastric conduit (Fig. 2). Both the patency of the vessels and perfusion of the conduit were confirmed. The following gastric conduit pull up through the retrosternal space and cervical esophagogastrostomy were performed without complications. The final pathologic report demonstrated a poorly differentiated squamous cell carcinoma (ypT2N0 M0).

**Fig. 3:**
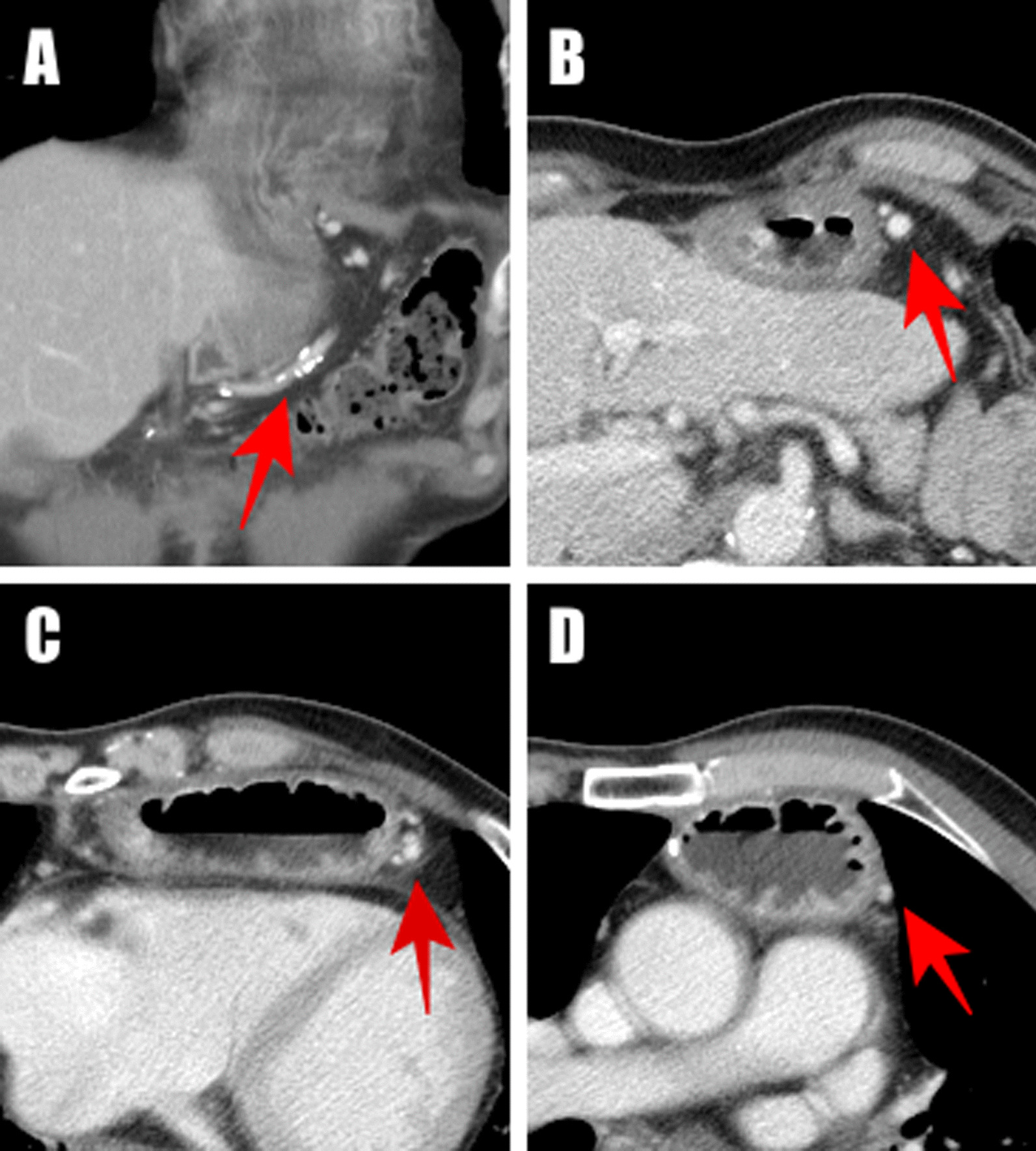
Postoperative contrast computed tomography scan at postoperative day 10 revealed a patent right gastroepiploic vessel (red arrow), which could be identified from the proximal (**A, B**) to the distal edge of the gastric conduit (**C, D**)

During the postoperative recovery period, no gastric conduit necrosis occurred. Esophagography was routinely performed on postoperative day 7 to evaluate the esophagogastrostomy, which did not disclose any extravasation of the contrast that was evident. However, some turbid fluid was found around the cervical incision on postoperative day 8, which suggested that a leakage of the esophagogastric anastomosis was occuring. It was treated successfully by conservative drainage and antibiotic administration. Follow-up computed tomography with contrast showed that there was no abscess around the leakage site. Meanwhile, the gastric conduit was fully vascularized and the right gastroepiploic artery was patent (Fig. 3). This patient was discharged at postoperative day 43 with normal oral intake. There were no subsequent complications related to malperfusion of the gastric conduit in the following 6 months either.

## Discussion and conclusions

Injury of the right gastroepiploic vessels during construction of a gastric conduit for esophagectomy is uncommon and its incidence rate remains unclear. Chen et al. [15]reported 3 cases of vascular reconstruction in 843 patients (0.36%) who underwent esophagectomy. The present case was the first case among 428 patients who underwent a McKeown esophagectomy between 2009 and 2018 in our institution; the incidence rate was 0.2%. In this patient, we repaired the injured vessels by microvascular anastomosis and subsequently performed the gastric conduit reconstruction with satisfactory final outcome.

Regarding our patient, we converted to a laparotomy immediately to explore and dissect the ends of the vessels after identifying the complication. The vascular stumps were clear-cut, and the pulsation of the proximal arterial stump could be visualized. The distance between the open ends of the transected vessels was measured to be approximately 2 mm, which suggested that a direct end-to-end anastomosis could be performed without tension. A preoperative computed tomography study demonstrated no atherosclerotic change in these vessels. These favorable conditions allowed us to perform a primary anastomosis for the injured vessels immediately. However, vascular reconstruction may not be feasible if there is a suspicion of inferior vascular quality, a long segmental injury, multiple sites of vessel injury or an excess increase in tension after anastomosis [15–17]. The latter is especially true for gastric conduit reconstruction because attention should be given to prevent torsion, tension and traction injuries of the repaired vessel during the pull-though of the gastric tube. It is worth mentioning that Chen et al.[15] recommended reconstructing the vessels in the vein-first order to prevent thrombosis in the cases with simultaneous RGEA and REGV injuries. Collectively, our findings suggest that vascular reconstruction for damaged right gastroepiploic vessels may serve as a feasible option to preserve the gastric conduit in selected patients.

Intraoperative identification of the injured vessels with prompt management is crucial to avoid immediate gastric conduit ischemia and the devastating complications stemming from the unawareness of this complication [7]. Some reports proposed that a jejunal or colonic interposition should be performed if the right gastroepiploic vessels are damaged during the harvest of the gastric conduit [3–5, 7, 8]. However, some reported series did not abandon the gastric conduit. Instead, they salvaged the gastric conduit by the intraoperative reconstruction of the damaged vessels [11–15]. A total of 7 cases were reported in these studies, which were found in an English literature search in the PubMed from database inception to May 2021. The details of the 7 cases plus the case in the present study are summarized in Table 1. Among these 8 cases, 4 of them (50%) had simultaneous RGEA and REGV injuries, 3 (37.5%) had an isolated RGEA injury and 1 (12.5%) had an isolated RGEV injury. A primary end-to-end anastomosis was performed in patients with simultaneous RGEA and REGV injuries, or an isolated RGEA injury. For the remaining patient with an isolated RGEV injury, a superdrainage of the gastric conduit was performed by anastomosing the omental vein to the pretracheal vein to avoid congestion. Additionally, similar to most reported cases, we converted the laparoscopic approach to a laparotomy and performed a microvascular anastomosis after trimming the injured vessels. Only one study[14] demonstrated a successful end-to-end anastomosis under robotic assistance without conversion to a laparotomy. Flow assessment of the re-anastomosed vessel is another key component to salvage the gastric conduit. The present study and two previous studies [12, 14] used the ICG fluorescence imaging technique during surgery to ensure the adequate perfusion of the gastric conduit and the patency of the reanastomosed vessels after vascular reconstruction. Alternatively, two other studies used the transit time ultrasound [11] and coronary blood flow measuring instruments [15] to evaluate the vascular patency during surgery. All the above mentioned studies performed gastric conduit for reconstruction after vascular reconstruction. Only one study [13] performed a staged reconstruction 6 days after the initial surgery owing to concerns about potential reperfusion tissue damage and traction injury to the reanastomosed vessels. Among the 8 cases in this review, 3 patients (37.5%) were reported to have an esophagogastrostomy leakage postoperatively. Instead of surgical interventions or resection of the conduit, all of these leakages were controlled conservatively. None of these 8 patients experienced gastric conduit failure at late postoperative period. Thus, the intraoperative reconstruction of the injured vessels with adequate flow assessment appears to be reliable for the immediate gastric conduit reconstruction in patients under this setting.

**Table 1:** Literature review of the reported cases with injury of right gastroepiploic vessel during esophagectomy and reconstruction

First Author	Year	Case no	Type of caner	Type of Esophagectomy	Type of gastric conduiting	Injured vessel	Management	Evaluation strategy for vascular patency	Outcome
Colon [11]	2016	1	GEJ adenocarcinoma	Ivor-Lewis	Laparotomy	RGEA	End-to-end anastomosis	Transit time ultrasound	Anastomosis leakage
Kitagawa [12]	2017	1	Esophageal SCC	N/A	N/A	RGEV	Venous superdrainage	ICG fluorescence imaging	No complication
van Boxel [13]	2020	1	GEJ adenocarcinoma	McKeown	Laparoscopy	RGEA + RGEV	End-to-end anastomosis	Staged reconstruction	No complication
Yun [14]	2020	1	GEJ adenocarcinoma	Ivor-Lewis	Robotic	RGEA	End-to-end anastomosis	Flourence ICG imaging	No complication
Chen [15]	2021	3	N/A	McKeown	N/A	RGEA + RGEV × 2 RGEA × 1	End-to-end anastomosis	Coronary blood flow measuring instrument	Anastomosis leakage × 1
Kou	2021	1	Esophageal SCC	McKeown	Laparoscopy	RGEA + RGEV	End-to-end anastomosis	Flourence ICG imaging	Anastomosis leakage

*GEJ* gastroesophageal junction, *SCC* squamous cell carcinoma, *RGEA* right gastroepiploic artery, *RGEV* right gastroepiploic vein, *ICG* indocyanine green, *N/A* not available

In conclusion, we reported a case of a successful restoration of an iatrogenically injured right gastroepiploic vessels using a microvascular anastomosis and an intraoperative ICG fluorescence assessment during gastric conduit harvest for esophagectomy. With timely identification of the injured vessels, intraoperative vascular reconstruction, and proper evaluation strategy of conduit perfusion, the above combined management may be considered as an option for patients encountering iatrogenic vascular compromise of the gastric conduit, instead of immediate conversion to other conduits for reconstruction.

## Data Availability

The dataset used and/or analyzed during the current study is available from the corresponding author on reasonable request.

## Abbreviations

RGEA: Right gastroepiploic artery
RGEV: Right gastroepiploic vein
ICG: Indocyanide green
